# Low Dose Growth Hormone Adjuvant Treatment With Ultra-Long Ovarian Stimulation Protocol in Poor Responders Showed Non-inferior Pregnancy Outcome Compared With Normal Responders

**DOI:** 10.3389/fendo.2019.00892

**Published:** 2019-12-20

**Authors:** Yi-Xuan Lee, Meng-Shun Shen, Chii-Ruey Tzeng

**Affiliations:** ^1^Graduate Institute of Clinical Medicine, Taipei Medical University, Taipei, Taiwan; ^2^Division of Infertility, Department of Obstetrics and Gynecology, Taipei Medical University Hospital, Taipei, Taiwan; ^3^IHMED Reproductive Medical Center, Taipei, Taiwan; ^4^Department of Obstetrics and Gynecology, School of Medicine, College of Medicine, Taipei Medical University, Taipei, Taiwan

**Keywords:** growth hormone, low dose, poor ovarian response, *in vitro* fertilization, adjuvant treatment, pregnancy outcomes

## Abstract

**Background:** Growth hormone (GH) has long been used as adjuvant treatment in ovarian stimulation for *in vitro* fertilization (IVF), especially in poor responder (PR) patients. However, its clinical efficacy remains unclear, and most studies are underpowered owing to their small sample size with different regimens.

**Methods:** Our study was divided into two parts. The first part was a parallel randomized, observational study in which 184 patients who fulfilled the criteria of poor ovarian response (POR) were enrolled and received ultra-long ovarian stimulation protocol with or without GH adjuvant therapy. For the second part, clinical data were retrospectively extracted from 163 patients classified as PRs who received 10 IU GH adjuvant therapy and 157 patients classified as normal responders (NRs) who received the same IVF protocol treatment without GH adjuvant therapy.

**Results:** For the first part of the study, the ovarian response, the number of oocytes retrieved, and the number of available embryos transferred were all significantly higher in the GH (+) group than in the GH (–) group. The clinical pregnancy rate was significantly higher in the GH (+) group (31.9 vs. 16.7%, *p* = 0.0168). The miscarriage rate did not differ significantly between the groups. The ongoing pregnancy rate was also significantly higher in the GH (+) group than in the GH (–) group (26.6 vs. 14.4%, *p* = 0.0418). Logistic regression revealed that the chance of clinical pregnancy in the GH (+) group significant increased 2.34-fold in comparison with the GH (–) group (*p* = 0.018). Subgroup analysis showed that the chance of clinical pregnancy in the GH (+) group significantly increased 2.38-fold (*p* = 0.034). The second part of the study showed no statistical difference between the PR with GH and the NR without GH groups regarding the implantation rate (15.6 vs. 19.8%, *p* = 0.3254) and the clinical pregnancy rate (31.9 vs. 39.5%, *p* = 0.1565). The NR without GH group showed insignificantly higher chance of clinical pregnancy (OR = 1.39, *p* = 0.157) compared with the PR with GH group.

**Conclusion:** Our results suggested that low-dose GH supplementation may improve ovarian response and pregnancy outcome in POR patients, particularly in patients younger than 40 years old. Moreover, the low-dose GH effect in POR patients resulted in non-inferior clinical pregnancy outcome compared with NRs.

## Introduction

Controlled ovarian hyperstimulation (COH) has long been a crucial part of *in vitro* fertilization (IVF), along with the development of assisted reproductive technologies (ART). The goal of COH is to recruit multiple follicles and to obtain many mature oocytes to increase chances for conception (1). Poor ovarian response (POR) to ovarian stimulation indicates a reduction in follicular response and a reduced number of retrieved oocytes. Despite using different stimulation protocols and multiple treatment courses of IVF, the pregnancy outcome remains poor in POR patients, which is frustrating for both the patients and the clinicians.

The incidencevs of POR to ovarian stimulation reportedly ranges from 9 to 24% of IVF-embryo transfer (ET) cycles, according to various studies (2, 3). POR, or poor responders (PRs), remains a significant challenge for IVF practice owing to not only the heterogeneity of the pathophysiology but also the lack of general consensus in the definition of POR. The latter directly leads to poor literature quality with insufficient evidence to identify any particular intervention to improve outcomes in POR patients. The definition of POR was under debate without uniform agreement for decades until the European Society of Human Reproduction and Embryology conducted a consensus study and reached a definition for POR in 2011. This so-called Bologna criterion defines poor response in IVF as comprising at least two of the following three features: (i) advanced maternal age (≥40 years) or any other risk factor for POR, (ii) previous POR (≤ 3 oocytes with a conventional stimulation protocol), and (iii) an abnormal ovarian reserve test [antral follicular count (AFC) < 5–7 follicles or anti-mullerian hormone (AMH) <0.5–1.1 ng/ml]. Two episodes of POR after maximal stimulation are sufficient to define a patient as a PR in the absence of advanced maternal age (4).

Numerous studies have been conducted using different interventions for the management of POR. Among them, the use of growth hormone (GH) as an adjuvant treatment with gonadotropins to facilitate follicular development and ovulation induction was first introduced by Homburg et al. in 1988 (5). GH is an anabolic peptide hormone which functions to increase cell growth and proliferation, and it has been reported to modulate the action of follicle-stimulating hormone (FSH) by binding to GH receptors on granulosa cells to increase the synthesis of insulin-like growth factor-I (IGF-I). IGF-I augments the effect of gonadotropin action on both the granulosa and theca cells and plays an essential role in follicular development, oocyte maturation, and steroidogenesis. It can also improve follicular survival and granulosa cell proliferation by directly inhibiting follicle apoptosis (5–9). Nevertheless, results from previous studies are controversial regarding the effect of GH as an adjuvant therapy during COH. GH has been demonstrated to increase the retrieved oocytes and improve embryo quality and pregnancy outcomes in several studies (10–19) and meta-analyses (2, 3, 20–22). However, several clinical trials failed to demonstrate significant benefits regarding clinical pregnancy and live birth rates (23–27). The small sample size of the trials, inconsistency in the definition of POR, and different stimulation protocols with GH regimens all contributed to the bias of the outcomes. Therefore, the true value of GH adjuvant treatment remains elusive to date.

Very few reports are available that discuss the influence of GH dosage and regimen. Furthermore, no previous study using an ultra-long down-regulation protocol combined with GH adjuvant treatment has been reported. In our study, we aimed to investigate the efficacy of low-dose GH adjuvant treatment in PR patients compared with normal responders (NRs) using an ultra-long ovarian stimulation protocol.

## Materials and Methods

### Study Period and Participants

This parallel randomized, observational cohort study was conducted in a single IVF center in Taipei Medical University Hospital from January 2010 to October 2012.

The study was divided into two parts. First, enrolled patients were classified as PRs who had fulfiled at least two of the following criteria: (i) advanced maternal age (≥ 40 years old) or any other risk factors for POR, (ii) previous episode of POR (≤ 3 mature oocytes retrieved with a conventional stimulation protocol), and (iii) an abnormal ovarian reserve test (AFC <5–7 follicles or AMH < 0.5–1.1 ng/ml) between January 2010 and November 2011. Second, the data of patients who were classified as PRs who received GH adjuvant therapy and as NRs who received the same IVF treatment protocol without GH adjuvant therapy were collected between January 2012 and October 2012.

### Clinical Management

To prevent possible bias from different physicians, all patients were handled by a single clinician. In addition, to eliminate bias from fresh vs. frozen embryo transfer, only the first IVF cycle with fresh embryo transfer (ET) within the study period was analyzed. All patients who participated in the study followed a gonadotropin-releasing hormone (GnRH) agonist ultra-long IVF protocol. In brief, the patients received a half-dose one-shot long-acting GnRH agonist (leuprolide acetate, 1.88 mg) at cycle day 1–3, followed by ovulation induction with gonadotropin starting between day 35 and 40. The dosage of gonadotropin was adjusted according to the ovarian response, which was monitored by transvaginal ultrasonography and serum hormone level. When two or more follicles reached a diameter of 17–18 mm, 6,500–10,000 IU of human chorionic gonadotropin (hCG) was administered, and transvaginal oocyte retrieval was performed 34–36 h later. Fertilization was conducted by intracytoplasmic sperm injection (ICSI) ~2 h later, after oocyte denudation. Fresh day 3 ET was performed with the best-quality blastomeres.

The first part of the study was a parallel, randomized study. Patients who fulfilled the abovementioned inclusion criteria were randomly allocated into two groups (named the GH (+) group and the GH (–) group) with simple randomization using a tossing coin method. Patients in the GH (+) group (*n* = 94) received co-treatment with GH adjuvant therapy (Saizen; Merck Serono) at a dosage of 4, 4, and 2 IU for three successive days, along with the ovulation induction. The total GH dosage was 10 IU for each patient in the GH (+) group. Patients in the GH (–) group (*n* = 90) received the same IVF protocol without GH adjuvant therapy.

For the second part of the study, patients who were classified as PRs and received co-treatment with GH adjuvant therapy (*n* = 163) and NR patients (*n* = 157) who received the same IVF protocol without GH adjuvant therapy were enrolled.

### Data Analysis and Statistics

Clinical parameters including age, AMH level, E2 level on the day of hCG administration, total gonadotropin dosage, mean number of oocytes retrieved, number of embryos transferred, embryo quality, and number of surplus embryos were recorded and analyzed. The main outcomes of the study were implantation rate, clinical pregnancy rate, miscarriage rate, and ongoing pregnancy rate. The implantation rate was calculated as the ratio of the number of gestational sacs to the number of embryos transferred. Clinical pregnancy was defined as the presence of a positive heartbeat in a healthy gestational sac, detected by transvaginal ultrasound 4–5 weeks after embryo transfer. Early miscarriage was defined as pregnancy loss before 12 weeks of gestation. Ongoing pregnancy was defined as viable pregnancy after 20 weeks of gestation.

Data are presented as mean and SD for quantitative variables and percentage for qualitative variables. The odds ratio (OR) and 95% confidence interval were calculated for clinical pregnancy rate and miscarriage rate. Comparisons between groups were performed using the Student's *t*-test for quantitative variables and the chi-square test for qualitative variables. SPSS (IBM Statistics, ver. 25) was used for all statistical analyses. A *p* < 0.05 was considered statistically significant.

## Results

### Outcomes of PR Patient With GH (+) and GH (–) Groups

The clinical parameters and main outcomes in both groups are listed in Table 1. The baseline characteristics of both groups including age and AMH did not differ statistically. The E2 level on the hCG day was significantly higher in the GH (+) group (679 ± 459 vs. 457 ± 357, *p* = 0.0003). The number of oocytes retrieved was also significantly higher in the GH (+) group than in the GH (–) group (5.5 ± 3.3 vs. 2.1 ± 0.7, *p* < 0.0001), both indicating a higher ovarian response to stimulation in the GH (+) group. The number of embryo transfer was significant higher in the GH (+) group than in the GH (–) group (2.6 ± 0.9 vs. 1.7 ± 0.7, *p* < 0.0001). The clinical pregnancy rate was significantly higher in the GH (+) group than in the GH (–) group (31.9 vs. 16.7%, *p* = 0.0168; OR = 2.34, *p* = 0.0177), and the number of ET cycles was significantly higher in the GH (+) group (2.6 ± 0.9 vs. 1.7 ± 0.7, *p* < 0.0001). The miscarriage rate did not differ significantly between the groups. The ongoing pregnancy rate was also significantly higher in the GH (+) group than in the GH (–) group (26.6 vs. 14.4%, *p* = 0.0418).

**Table 1:** Clinical parameters and outcomes of poor-responders with GH (+) and GH (–) groups.

	**GH (+)**	**GH (–)**	***P*-value**
*N*	94	90	
AMH, ng/ml	1.1 ± 0.4	1.1 ± 0.9	1.0000
Age, year	38 ± 3.4	37.1 ± 3.8	0.0919
E2 level on hCG day, pg/ml	679 ± 459	457 ± 357	0.0003^*^
No. of oocytes retrieved	5.5 ± 3.3	2.1 ± 0.7	<0.0001^*^
No. of embryos transfer	2.6 ± 0.9	1.7 ± 0.7	<0.0001^*^
Clinical pregnancy, *n* (%)	30/94 (31.9%)	15/90 (16.7%)	0.0168^*^
Miscarriage, *n* (%)	5/30 (16.6%)	2/15 (13.3%)	0.7755
Ongoing pregnancy, *n* (%)	25/94 (26.6%)	13/90 (14.4%)	0.0418^*^

*All values presented as mean ± SD, unless stated otherwise*.

* *p < 0.05, statistically significant*.

We further subdivided both patient cohorts by age below and above 40 years old. The results showed that the E2 level on the hCG day was significantly higher in the GH (+) group (619 ± 317 vs. 449 ± 366, *p* = 0.0065) in patients under 40 years old but not in patients older than 40 years (685 ± 408 vs. 503 ± 336, *p* = 0.0716). Logistic regression revealed that the chance of clinical pregnancy in the GH (+) group significantly increased 2.34-fold (*p* = 0.018) in univariable analysis and 2.52-fold (*p* = 0.011) in multivariable analysis, respectively (Table 2). Subgroup analysis showed that the chance of clinical pregnancy in the GH (+) group significantly increased 2.38-fold (*p* = 0.034) in patients <40 years old, but not in patients more than 40 years old (OR = 3.23, *p* = 0.17; Table 3).

**Table 2:** Logistic regression with univariate and multivariate analysis of the pregnancy outcome for poor-responders.

**Variable**		**Clinical pregnancy rate, *N* (%)**	**Clinical pregnancy OR (95% CI)**			
			**Univariable analysis (Crude OR)**	***P*-value**	**Multivariable analysis (Adjusted OR)**	***P*-value**
Growth Hormone	GH (+)	30 (31.9%)	2.34 (1.16–4.74)	0.018^*^	2.52 (1.23–5.16)	0.011^*^
	GH (–)	15 (16.7%)	1.00		1.00	
Age	<40	36 (28.8%)	2.25 (1.00–5.04)	0.05	2.46 (1.08–5.62)	0.032^*^
	≥40	9 (15.2%)	1.00		1.00	

* *p < 0.05*.

**Table 3:** Subgroup analysis of the pregnancy outcome for poor-responders.

**Variable**	**Subgroup**	**Clinical pregnancy rate, *N* (%)**	**Clinical pregnancy OR (95% CI)**	***P*-value**
GH (+)	Age <40	23 (37.7%)	2.25 (0.84–6.03)	0.106
	Age ≥ 40	7 (21.2%)	1.00	
GH (–)	Age <40	13 (20.3%)	3.06 (0.63–14.64)	0.162
	Age ≥ 40	2 (7.7%)	1.00	
Age <40	GH (+)	23 (37.7%)	2.38 (1.07–5.28)	0.034^*^
	GH (–)	13 (20.3%)	1.00	
Age ≥ 40	GH (+)	7 (21.2%)	3.23 (0.61–17.10)	0.17
	GH (–)	2 (7.7%)	1.00	

* *p < 0.05*.

### Outcomes of PR Patients With GH and NR Patients Without GH

We compared the clinical parameters and main outcomes of PRs with GH co-treatment and NRs without GH co-treatment (Table 4). The PR with GH [PR + GH (+)] patients were significantly older than the NR without GH [NR + GH (–)] patients (38.8 ± 4.1 vs. 35 ± 3.8, *p* < 0.0001), with significantly lower AMH (1.6 ± 1.4 vs. 3.6 ± 2.7, *p* < 0.0001). During ovulation induction, the PR + GH (+) patients required higher total gonadotropin (2495 ± 915 vs. 2160 ± 513, *p* = 0.0001), but had lower E2 level on hCG day (872 ± 723.6 vs. 1652 ± 1141, *p* = 0.0001) compared with the NR + GH (–) patients. The number of oocytes retrieved, the number of good-quality embryos, and the number of surplus frozen embryos were all significantly lower in the PR + GH (+) group than in the NR + GH (–) group. However, there was no statistical difference between the two groups regarding the number of embryos transferred (2.5 ± 1.0 vs. 2.6 ± 1.8, *p* = 0.5375), the implantation rate (15.6 vs. 19.8%, *p* = 0.3254), and the clinical pregnancy rate (31.9 vs. 39.5%, *p* = 0.1565).

**Table 4:** Clinical parameters and outcomes of poor-responder with GH co-treatment and normal-responder without GH co-treatment groups.

	**PR with GH (+)**	**NR with GH (–)**	***P*-value**
*N*	163	157	
Age, years	38.8 ± 4.1	35 ± 3.8	<0.0001^*^
AMH, ng/ml	1.6 ± 1.4	3.6 ± 2.7	<0.0001^*^
Total FSH, IU	2495 ± 915	2160 ± 513	0.0001^*^
E2 level on hCG day, pg/ml	872 ± 723.6	1652 ± 1141	0.0001
No. of oocytes retrieved	5.8 ± 4.1	10.3 ± 6.0	<0.0001^*^
No. of good embryos	3.8 ± 2.7	7.0 ± 4.3	<0.0001^*^
No. of embryos transfer	2.5 ± 1.0	2.6 ± 1.8	0.5375
No. of surplus embryos frozen	0.7 ± 1.7	3.0 ± 3.8	<0.0001^*^
Implantation rate	15.6%	19.8%	0.3254
Clinical pregnancy rate, *n* (%)	52/163 (31.9%)	62/157 (39.5%)	0.1565

* *p < 0.05*.

Logistic regression revealed that the chance of clinical pregnancy in patients <40 years old significantly increased 2.41-fold (*p* = 0.003) in univariable analysis and 2.33-fold (*p* = 0.006) in multivariable analysis, respectively (Table 5). The NR + GH (–) group showed a slightly higher chance of clinical pregnancy (OR = 1.39, *p* = 0.157 in univariable; OR = 1.08, *p* = 0.76 in multivariable analysis, respectively), but these differences were insignificant (Table 5). Subgroup analysis showed that the chance of clinical pregnancy in the GH (+) group significantly increased 2.13-fold (*p* = 0.033) in patients <40 years old compared with patients more than 40 years old. The chance of clinical pregnancy showed an insignificant increase in patients <40 years old (OR = 1.14, *p* = 0.63) and an insignificant decrease in patients more than 40 years old (OR = 0.78, *p* = 0.72) (Table 6).

**Table 5:** Logistic regression with univariate and multivariate analysis of pregnancy outcome for poor-responder (PR) with GH co-treatment and normal-responder (NR) without GH co-treatment groups.

**Variable**		**Clinical pregnancy rate, *N* (%)**	**Clinical pregnancy OR (95% CI)**			
			**Univariable analysis (Crude OR)**	***P*-value**	**Multivariable analysis (Adjusted OR)**	***P*-value**
Growth Hormone	PR (*N* = 163) GH (+)	52 (31.9%)	1.00		1.00	
	NR (*N* = 157) GH (–)	62 (39.5%)	1.39 (0.88–2.20)	0.157	1.08 (0.66–1.77)	0.76
Age	<40 (*N* = 234)	95 (40.6%)	2.41 (1.36–4.27)	0.003^*^	2.33 (1.27–4.29)	0.006^*^
	≥40 (*N* = 86)	19 (22.1%)	1.00		1.00	

* *p < 0.05*.

**Table 6:** Subgroup analysis of the pregnancy outcome for poor-responder (PR) with GH co-treatment and normal-responder (NR) without GH co-treatment groups.

**Variable**	**Subgroup**	**Clinical pregnancy rate, *N* (%)**	**Clinical pregnancy OR (95% CI)**	***P*-value**
PR, GH (+), *N* = 163	Age <40	36 (38.7%)	2.13 (1.06–4.28)	0.033^*^
	Age ≥ 40	16 (22.9%)	1.00	
NR, GH (–), *N* = 157	Age <40	59 (41.8%)	3.12 (0.85–11.43)	0.086
	Age ≥ 40	3 (18.8%)	1.00	
Age <40, *N* = 234	PR, GH (+)	36 (38.7%)	1.00	0.63
	NR, GH (–)	59 (41.8%)	1.14 (0.67–1.95)	
Age ≥ 40, *N* = 86	PR, GH (+)	16 (22.9%)	1.00	0.72
	NR, GH (–)	3 (18.8%)	0.78 (0.20–3.08)	

* *p < 0.05*.

## Discussions

The first part of our study demonstrated that, in POR patients, low-dose GH supplementation significantly increased ovarian response, the number of oocyte retrieved, clinical pregnancy rate, and the ongoing pregnancy rate. The number of embryo transferred was also significant increase, which was mainly due to more available embryos obtained after GH supplementation. These results echoed the conclusions of previous studies and several meta-analyses which reported increased ovarian response and pregnancy outcome after GH supplementation. Using logistic regression and subgroup analysis, our results demonstrated that the effect of GH seemed more prominent in younger patients (<40 years old).

In the second part of our study, the POR patients were assumed to have poor prognosis because they were apparently older and had a significantly lower ovarian reserve than the NRs. POR patients with low-dose GH supplementation required a higher gonadotropin dosage but ended up with lower ovarian response, smaller oocyte yields, fewer good-quality embryos, and fewer surplus frozen embryos. However, the implantation rates and clinical pregnancy rates were comparable between POR patients with low-dose GH adjuvant treatment and NRs without GH.

GH exerts biological effects on most of the tissue, including tissue metabolism and induced local synthesis of IGF-I to facilitate cell growth (8). In ovarian function, GH is necessary for oogenesis and folliculogenesis, which is fundamental for optimal female fertility (28). In the second part of study, we believed that the effect of GH compensated the poor-prognosis of this group and brought about comparable, or at least non-inferior, pregnancy outcomes compared with the NRs. Nevertheless, we could not rule out the beneficial effect of GH on other aspects, for example, the endometrium, that influenced pregnancy outcomes, since the NR group still possessed more oocytes and a higher embryo yield. The uterus is also a site of both GH production and GH action (28). The glandular cells of human endometrium and decidual tissue express GH receptors from the late luteal phase throughout pregnancy. Therefore, GH is speculated to play an important role in implantation (29). Previous studies also reported that GH might improve clinical outcome by increasing endometrial blood perfusion and improving endometrial receptivity during frozen-thawed ET in patients with repeated implantation failure (30, 31).

There is no standard protocol regarding GH regimen and dosage to date. The use of GH ranges from 4 to 24 IU, depending on different studies, and is usually started from previous cycle day 21 until the day of hCG administration. It is usually injected daily or on alternate days (2, 20, 21). Most studies have shown a positive impact of GH on ovarian response and pregnancy outcome, without adverse effect reported. Nevertheless, GH may have a detrimental effect on insulin resistance, and high GH levels can inhibit fertility and promote neoplasm; the exact threshold dosage is still unclear (28). Moreover, an increased economic burden is inevitable since GH is very expensive. To our best knowledge, ours is only the second low-dose GH supplementation study in the literature. Lattes et al. (14) conducted a self-controlled study of 64 PRs who failed to reach pregnancy in the previous cycle and were the first to use a GnRH agonist long protocol co-treatment with low-dose GH supplementation (0.5 IU/day) from previous cycle day 21 until the day of hCG injection. The average day of COH was 11.2 days, and the estimated total dose of GH was about 9.5–10 IU, almost the same as in our study. However, in addition to a different study design, the regimen we used required injections for only 3 days. Furthermore, we used an ultra-long GnRH agonist protocol, which is less common worldwide. In fact, we believe that the ultra-long protocol is effective, flexible, and more patient-friendly when combined with long-acting gonadotropin to reduce the injection times.

We acknowledged that the limitations of our studies included a small sample size and the retrospective nature of the second part of the study. In addition, our results did not take into account the condition of the endometrium and other clinical parameters such as ploidy status, fertilization rate, and live birth rate. A randomized prospective clinical trial with a larger sample size is needed to validate the preliminary results.

In conclusion, our results suggested that low-dose GH supplementation may improve oocyte and embryo yields, clinical pregnancy rate, and ongoing pregnancy rate in POR patients, especially in patients younger than 40 years old. The effect of low-dose GH in POR patients resulted in non-inferior clinical pregnancy outcomes compared with NRs.

## Data Availability Statement

The datasets generated for this study are available on request to the corresponding author.

## Ethics Statement

The study involving human participants was reviewed and approved by the Joint Institutional Review Board of Taipei Medical University (TMU-JIRB, approval number: N201505056) for retrospective analysis and clinical data reporting. TMU-JIRB waived the requirement for further specific ethics approval or written informed consent since all procedures were not altered from routine clinical protocols, in accordance with the national legislation and the institutional requirements.

## Author Contributions

The present work was designed by C-RT. Data extraction and analysis were performed by Y-XL and M-SS. Patient recruitment was undertaken by C-RT. The initial manuscript draft was prepared by Y-XL and subsequently revised by Y-XL and C-RT. All authors approved the final submitted version.

### Conflict of Interest

The authors declare that the research was conducted in the absence of any commercial or financial relationships that could be construed as a potential conflict of interest.
